# 2715. Toxicities and clinical consequences of valganciclovir prophylaxis in solid organ transplant recipients

**DOI:** 10.1093/ofid/ofad500.2326

**Published:** 2023-11-27

**Authors:** Chloe Lahoud, Andres E Franceschi, Fabiola Reyes, Dimitrios G Moshovitis, Angela Achkar, Antara Chakraborty, Manu Balusu, Jawad Safiia, Eliezer Zachary Nussbaum, Alyssa R Letourneau, Sarah P Hammond, Camille N Kotton, Sophia Koo

**Affiliations:** Staten Island University Hospital, Staten Island, New York; Brigham and Women's Hospital, Brookline, Massachusetts; Brigham and Women's Hospital, Brookline, Massachusetts; Brigham and Women's Hospital, Brookline, Massachusetts; Post-Doctoral research fellow, Boston, Massachusetts; Brigham and Women's Hospital, Brookline, Massachusetts; Brigham and women's hospital, Boston, Massachusetts; Brigham and Women's Hospital, Brookline, Massachusetts; Tufts Medical Center/Tufts University School of Medicine/Division of Geographic Medicine and Infectious Disease, Cambridge, Massachusetts; Massachusetts General Hospital, Boston, Massachusetts; Massachusetts General Hospital, Boston, Massachusetts; Massachusetts General Hospital, Boston, Massachusetts; Brigham and Women's Hospital, Dana-Farber Cancer Institute, Boston, Massachusetts

## Abstract

**Background:**

Cytomegalovirus (CMV) commonly affects solid organ transplant (SOT) recipients, with increased risks of rejection, graft failure, and other infections. While valganciclovir (vGCV) prophylaxis is effective in preventing CMV, its use is sometimes hindered by toxicity. We examined the association between vGCV exposure and adverse outcomes in SOT recipients.

**Methods:**

We performed a retrospective cohort study in two large academic transplant centers in Boston, with 1650 sequential adult (≥ 18 years of age) lung (343), heart (200), and kidney (1107) transplant recipients from 2010-2016. CMV D+ and/or CMV R+ SOT recipients generally received 3-12 months of vGCV prophylaxis. We used Fisher’s exact tests, Wilcoxon rank-sum tests, and Cox models to examine the relationship between vGCV exposure and adverse events potentially related to vGCV exposure.

**Results:**

Median age was 56 (IQR 44, 64) years, 1040 (63%) were male, median BMI was 26.8 (23.1, 30.5) kg/m^2^, 594 (36%) were CMV D+R-, 397 (24%) D+R+, 333 (20%) D-R+, and 319 (19%) D-R-. Total vGCV exposure in patients receiving prophylaxis was 182 (IQR 108, 258) days. Patients receiving vGCV prophylaxis were significantly more likely to develop incident leukopenia with a white blood cell (WBC) count < 4000 cells/μL (**Figure**), severe leukopenia with a WBC < 1500 cells/μL (logrank p< 0.001), and incident thrombocytopenia < 150,000/μL (< 0.001) compared to patients not receiving vGCV. Early vGCV discontinuation, treatment interruption, reductions of vGCV below the dose appropriate for their renal function, and discontinuation or interruption of trimethoprim-sulfamethoxazole (TMP-SMZ) and mycophenolate due to myelosuppression were common, and patients exposed to vGCV were more likely to require G-CSF (**Table 1**). Opportunistic infections during holding of TMP-SMZ or vGCV, incident CMV resistance mutations, and rejection events occurring when mycophenolate was held for myelosuppression were extremely rare. After adjusting for age, BMI, and SOT type, vGCV exposure remained strongly associated with the risk of incident leukopenia (**Table 2**).

**Table 1.**
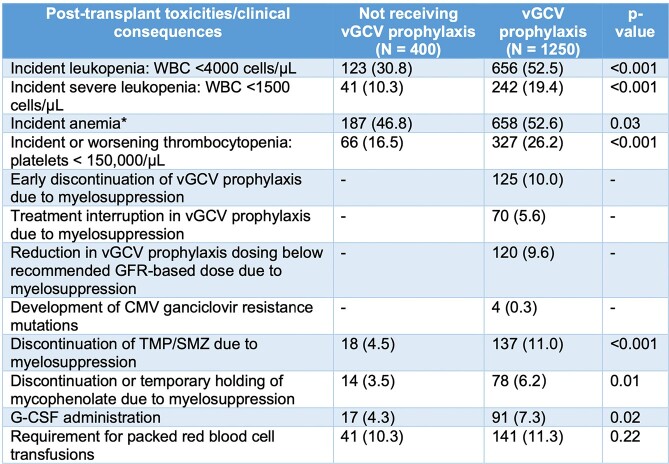
Potential toxicities associated with vGCV exposure. *Hemoglobin < 12 g/dL for women and < 13.5 g/dL for men or worsening anemia (drop >1 g/dL from baseline chronic anemia).

**Figure:**
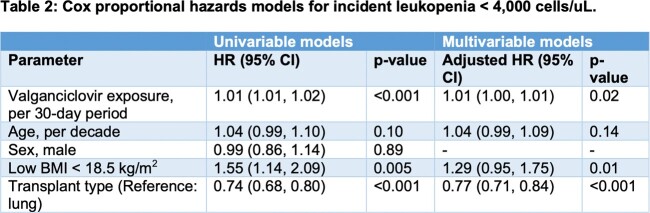

**Figure.**
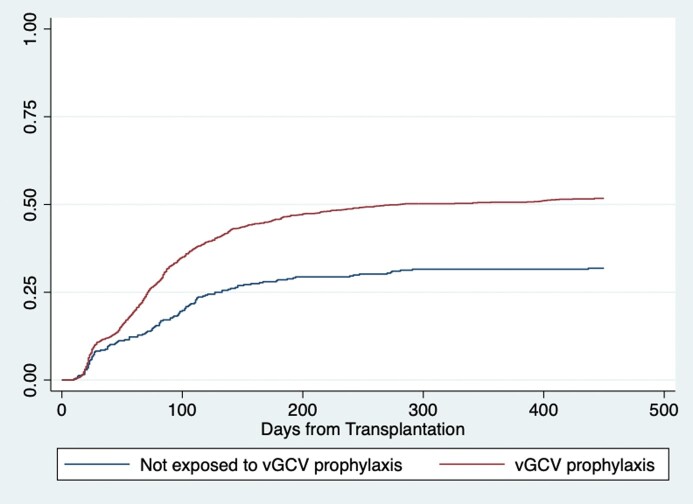
All transplant recipients, Kaplan-Meier cumulative incidence of leukopenia < 4,000 cells/uL (logrank p < 0.001).

**Conclusion:**

Exposure to vGCV prophylaxis in SOT recipients is associated with high rates of hematologic toxicities, although downstream clinical consequences of these toxicities are relatively rare.

**Disclosures:**

**Sarah P. Hammond, MD**, F2G: Advisor/Consultant|F2G: Grant/Research Support|GSK: Grant/Research Support|Pfizer: Advisor/Consultant|Scynexis: Grant/Research Support|Seres therapeutics: Advisor/Consultant **Sophia Koo, MD, SM**, Aerium Therapeutics: Advisor/Consultant|GSK: Grant/Research Support|Merck: Grant/Research Support|Scynexis: Grant/Research Support

